# Effectiveness of Bilateral Superficial Cervical Plexus Block as Part of Postoperative Analgesia for Patients Undergoing Thyroidectomy in Empress Zewditu Memorial Hospital, Addis Ababa, Ethiopia

**DOI:** 10.1155/2018/6107674

**Published:** 2018-01-21

**Authors:** Zemedu Aweke, Wosenyeleh A. Sahile, Sileshi Abiy, Nugusu Ayalew, Adugna A. kassa

**Affiliations:** ^1^Dilla University, Dila, Ethiopia; ^2^Addis Ababa University, Addis Ababa, Ethiopia

## Abstract

**Introduction:**

The pain after thyroid surgery is considered of moderate intensity and short duration. Most trials showed significant reduction in pain intensity and severity of pain in patients for whom bilateral superficial cervical plexus block (BSCPB) was done.

**Objective:**

To assess the postoperative analgesic effect of BSCPB for thyroid surgery.

**Methods:**

Sixty six euthyroid patients were recruited and assigned to two groups (33 patients each). Group 1 BSCPB and Group 2 standard analgesia. The unpaired Student's *t*-test and Mann–Whitney test were used for comparison. Statistical significance was stated at *p* value < 0.05.

**Results:**

The median postoperative pain score (NRS) was 3 in the BSCPB group and 5 in the control group (*p*=0.002). There was also statistically significant difference at 6th, 12th, and 24th hour showing a lower median pain score in the BSCPB group compared to the control group. The median time was (360 minutes) in the treatment group and (180 minutes) in the control group (*p*=0.0006). The median tramadol consumption within 24 hours is 0 mg in the BSCPB group compared to 100 mg in the control group (*p*=0.001).

**Conclusion and Recommendation:**

BSCPB done for thyroidectomy under general anesthesia decreases the postoperative pain score, total analgesia consumption, and time to first analgesia request.

## 1. Background

The total goiter prevalence in the global population is estimated to be 15.8%, and the highest prevalence of 28.3% is observed in Africa [1]. It is estimated that half of the Ethiopian population faces iodine deficiency, where 14 million or 40% of those at risks are believed to have goiter. The proportion of Addis Ababa households who consume iodized salt is 30%, which is higher compared to that in rural areas (13%). Though goiter with iodine deficiency is treated with iodine supplementations, goiter that does not regress in size, rebound growth, and presence of pressure symptoms are among indications for surgical treatment [2].

Anesthesia for thyroid surgeries is commonly done under general anesthesia. A mean postoperative pain score of 69 mm was reported on a 100 mm Visual Analogue Scale (VAS). It has also been reported that the morphine consumption in the first postoperative day is 90%. Studies also show that the proportion of patients with a pain score greater than 40 mm is 70% on the VAS scale [3, 4]. Cervical plexus block, either superficial or deep or combinations given bilaterally, could easily lead to adequate block appropriate for thyroid surgery without any significant side effects [5, 6]. It is associated with decreased requirement of opioids and lesser complications like postoperative nausea and vomiting, postoperative pulmonary complications, and longer hospital stay. Cervical plexus block has also been effectively used in other surgeries like carotid endarterectomy and lymph node biopsy/excision [7, 8].

BSCPB is also known for decreasing intraoperative analgesic requirements when given before surgery [3, 9]. By reducing analgesic requirements, the block yields stable operative conditions compared to general anesthesia alone. Studies have gone as far as recommending to use BSCPB as a sole anesthetic technique for smaller anterior neck surgeries for patients with comorbidities. BSCPB is also easier and safer than the combined superficial and deep cervical plexus block [10, 11]. It has also showed increased analgesic quality when compared with local infiltration [12].

Besides decreasing cost and side effects of opioids, the use of BSCPB also supports the principle of multimodal analgesia where a variety of analgesic medications given together might have additive or synergistic effects and more effective pain relief when compared with single-modality interventions. The objective of this study is hence to assess analgesic effectiveness of bilateral superficial cervical plexus block (BSCPB) for postthyroidectomy pain control.

## 2. Methodology

After ethical approval was obtained from Addis Ababa University Ethical Committee, all patients who underwent thyroid surgery from December 20, 2016, to May 30, 2017, were included in the study. Patients with substernal goiter, age <18, emergency reoperation, and preexisting neck pain and patients with respiratory disease were excluded from the study. Sampling for each group was done based on two independent sample size formulae relying on the mean difference of the VAS score, time to first analgesia request, and total analgesia request among the two groups. A total of 35 ASA I and ASA II euthyroid patients were assigned to each group. Afterwards, all patients who were scheduled for elective thyroidectomy were enrolled in the study and assigned to either the BSCPB or control group randomly. Four patients were found to have incomplete data and were removed from the study.

### 2.1. Superficial Cervical Plexus Block

The cervical plexus is derived from the C1, C2, C3, and C4 spinal nerves and supplies branches to the vertebral muscles, strap muscles of the neck, and phrenic nerve. Blockade of this distribution results in anesthesia of only cutaneous branches. The block is relatively easy to perform and provides anesthesia in the areas innervated with C2-C4-like lymph node dissections and carotid endarterectomies. If used bilaterally, it can also be used for thyroidectomies.

After preoperative preparation, all patients who were scheduled for elective thyroidectomy, who fulfilled inclusion criteria, and who volunteered to take part in the study were instructed on how to self-report pain using the eleven-point NRS score 0 to 10 in the morning of the operation day at ward with a trained nurse. On the day of the surgery, all anesthesia management was carried out by the assigned bachelor's and master's anesthetists.

Patients were sedated with 2 mg midazolam and 10 mcg fentanyl before the procedure. After cleaning the skin with an antiseptic solution, a hypodermic needle is inserted along the posterior border of the sternocleidomastoid muscle, landmark was identified as the midline between the mastoid process and clavicular head of the sternocleidomastoid muscle, and three injections of 5 mL of local anesthetic were done behind the posterior border of the sternocleidomastoid muscle subcutaneously, perpendicularly, cephalad, and caudad in a fan fashion. As a standard of care, patients were given 10 mg dexamethasone during induction to prevent surgery related to postoperative nausea and vomiting. Afterwards, standard anesthesia management with endotracheal intubation and monitoring were maintained.

At PACU, patients were asked to report their pain based on the 11-point NRS score as soon as the patient starts to fully respond to verbal command. The NRS score and other variables were documented at 3rd hour, 6th hour, 12th hour, and 24th hour at wards after the end of surgery. A time in minutes from the end of surgery to the first analgesia request was documented together with total analgesia consumed in the first 24 hours. In addition, the incidence of postoperative nausea and vomiting was also documented when it was reported within 24 hours.

Statistical analysis was done using the SPSS version 20 software. The Shapiro–Wilk test was used to test for distributions of data, while homogeneity of variance was assessed using Levene's test for equality of variance. Comparison of numerical variables between study groups was done using the unpaired Student's *t*-test and Mann–Whitney test. The frequency and percentage were used to describe categorical variables, and the statistical difference between groups was tested using the chi-square test. A *p* value < 0.05 with a power of 80% was considered statistically significant.

## 3. Results

### 3.1. Demographic and Perioperative Characteristics

A total of 66 patients (33 on each group) were involved in the study. There was no statistical difference between two groups in age with a *p* value of 0.429. The majority of study participants were female owing to the higher incidence of thyroid disease in females, but there is no statistical difference between two groups. The demographic status and peri-induction data were comparable between two groups with a *p* value greater than 0.05 as shown in Table 1.

### 3.2. Postoperative Recovery Room Vital Sign

The baseline vital signs (PR, SBP, DBP, MAP, and SPo2) taken in the recovery room before any medication was given were comparable between two groups with the exception of the heart rate (Table 2).

### 3.3. Comparison of Total Intraoperative Analgesia Consumption between Groups

Tramadol and diclofenac were used as intraoperative supplementation based on secondary data obtained from the anesthesia chart. The median and interquartile range between groups are shown in Table 3.

### 3.4. Comparison of Postoperative Numeric Pain Rating Scale

**Figure 1:**
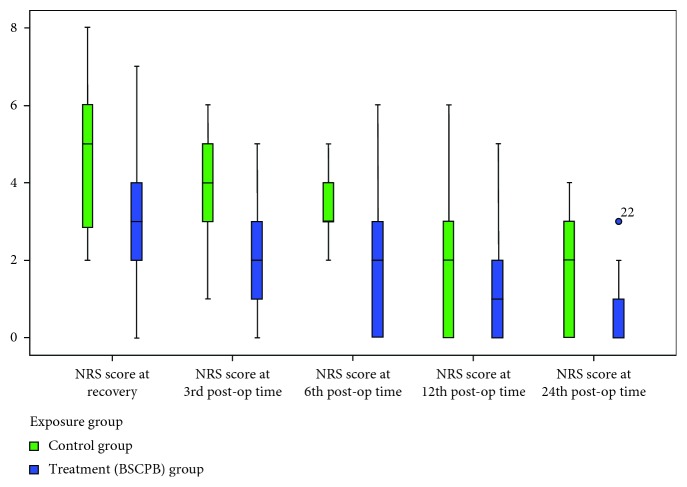
Comparison of postoperative pain using the 11-point NRS score (0–10).

The median NRS score was lowest in the block group at recovery room, 3rd, 6th, 12th, and 24th hour. Using the Mann–Whitney test, a significant statistical difference was observed at all time between block and control groups (*p* < 0.05) (Figure 1).

### 3.5. Comparison of Time to First Analgesia Request and Total Analgesia Consumption

There was a statistically significant difference in regard to median time to first analgesia request in minutes between the two groups as well as total tramadol consumption within 24 hours but no difference in total diclofenac consumption within 24 hours (Table 4).

### 3.6. Incidence of Nausea and Vomiting

**Figure 2:**
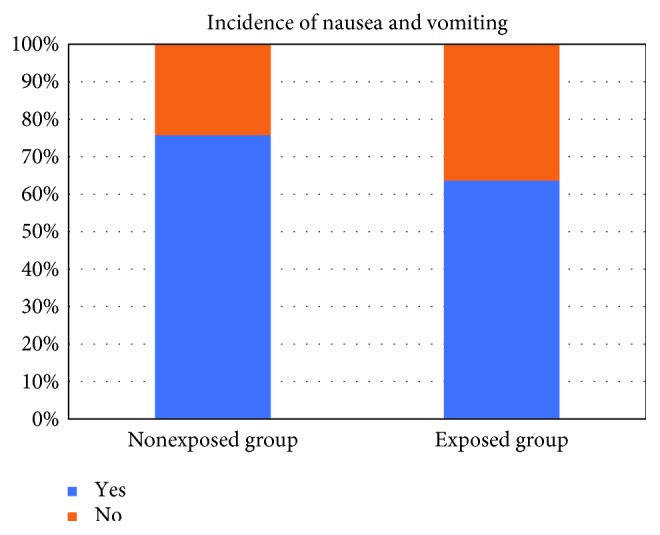
Incidence of nausea and vomiting between two groups.

The incidence of nausea and vomiting over 24 hours is 69.7%. The proportion of patients with nausea and vomiting is lower (63.63%) in the block group (BSCPB) compared to the control group which is 75.7% with a *p* value of 0.42 (Figure 2).

## 4. Discussion

The main finding of the study was the lower pain scores in the block group with a median (IQR) pain score of 3 (2–4) compared to 5 (3–6) in the control group with a *p* value of 0.002 at the immediate recovery room. The median NRS score at 3rd postoperative time is lower for the block group 2 (1–3) compared to 4 (3–5) in the control group (*p* < 0.0001). The median postoperative pain score was also lower at 6th, 12th, and 24th postoperative time with a statistically significant difference of <0.0001, 0.004, and <0.0001, respectively.

The result of this study is in line with the study done in France showing the lower pain score in the block group compared to the control group. This randomized controlled trial demonstrates that the median (IQR) pain score in the treatment group is 3 (0–10) and 5 (0–8) in the control with placebo group, respectively, *p*=0.01 [12]. The likely explanation for the similarity between two studies is the performance of the block, which was done after induction of anesthesia in both studies. Though the latter one uses ropivacaine 0.487%, we did not notice a significant difference in the immediate recovery room (PACU) pain score difference due to the difference in medication used. But in contrast to such a finding, a study done in Turkey did not show efficacy of the block. The mean VAS score at first hour was 23 + 19.3 in the BSCPB group compared to 20.7 + 13.3 in the control group with 0–100 VAS (*p* > 0.05) [13]. The possible explanation for this contradictory result is the difference in study design and pain management practice in the study setup.

The proportion of patients who had a NRS score greater than 4 at any time during 24 hours was 40.9% (30.3% control group, 10.6% block group), with a *p* value of 0.003. As shown by the Dieudonne et al. study, the proportion of patients who require additional morphine in the recovery room is 69%, where 39.1% of them were from the placebo group and 29.9% were from the bupivacaine group with a *p* value of 0.006. Though the same assessment tool NRS was used, adherence of patients to NRS between two populations may be attributed to the proportion difference observed [14].

The total postoperative tramadol consumption in the study was lower in the block group. The median (IQR) tramadol was 0 (0–50) mg in the block group compared to 100 (25–150) mg in the control group with *p*=0.001. Such a finding is comparable with the Turkey study which shows that median tramadol consumption was lower in the treatment group compared to the control group, 0 (0–50) versus 40 (0–180) mg, respectively, *p* < 0.05 [15]. Though different drugs were used, a study done in France reveals that total postoperative morphine consumption in the bupivacaine group is lower than that of the control group with median (range) 6 mg (2–39) compared to 12 mg (2–39). The use of BSCPB before surgery also decreased the total tramadol consumption within the first 24-hour period 0 mg (0–50 mg) versus 100 mg (50–150 mg) with a *p* value of 0.001. Though our study used tramadol. By using the opioid conversion factor suggested in studies, a 100 mg of tramadol could deliver equal analgesic potency as 10 mg of morphine. Hence a comparable result [14, 16].

We also observed that the median (IQR) of total diclofenac consumption within 24 hours was not statistically significant between block and control groups (75 mg (0–75 mg) versus 75 mg (0–75 mg), resp. (*p*=0.775)). We couldnt verify our findings because most studies only use opioids for postoperative pain management in both treatment and control groups. Thus, lack of the standard postoperative pain management protocol in the study hospital was among the possible factors for the similarity of diclofenac consumption between groups.

With regard to the time to first analgesia request, the finding showed a significant difference between the groups. The median (IQR) minute is 360 (190–720) versus 180 (65–360) between block and control groups, respectively (*p*=0.006). The result is comparable with a study done in Taiwan with a median time of 410.1 9 (15–1050) minutes in the treatment group with levobupivacaine and 360.8 (15–870) minutes in the treatment group with bupivacaine 0.5% longer than the placebo group with NS 82.1 (15–259) minutes. The median time in minutes required for analgesia request was higher in bupivacaine and levobupivacaine groups compared to saline having a significant *p* value of 0.0004 [17].

The overall incidence of nausea and vomiting within 24 hours was found to be 69.7%. This proportion is higher in the control group with an incidence of 75.7% compared to 63.63% in the treatment group. Though there is a proportion difference, there is no statistical difference between two groups (*p*=0.422). The findings are higher compared to Andrieu et al. study where the incidence of PONV is 36% [13]. The likely explanation for this incongruity would be that Andrieu et al. used premedication with hydroxyzine and used propofol as a standard induction agent which is known for its prophylaxis for nausea and vomiting.

The main limitations of this study are as follows: lack of randomization and control over the confounding factor even though most variables are comparable between groups; variability in the performance of the BSCPB since different anesthetists were involved; lack of the standard pain management protocol in the study hospital; and use of secondary data for preoperative and intraoperative variables. The main strength could be the homogeneity between the two groups.

As a summary, the result of our study demonstrates that bilateral superficial cervical plexus block (BSCPB) performed after induction of anesthesia with 0.25% bupivacaine is an effective and useful technique for postoperative analgesia for thyroid surgery patients. We recommend that BSCPB be considered as a primary analgesic method for such patients.

## Figures and Tables

**Table 1:** Demographic and intraoperative characteristics of patients.

	Treatment group (BSCPB) (*n* = 33)	Control group (*n* = 33)	*p* value
Age (years)^∗^	30 (10)	32 (20)	0.429
Sex (M/F)	5/28	3/30	0.708
ASA status			0.427
ASA I (*n*, %)	31 (47%)	28 (42%)	
ASA II (*n*, %)	2 (3%)	5 (8%)	
Preoperative diagnosis			0.424
Benign mass (*n*, %)	31 (47%)	28 (42%)	
Neoplastic mass (*n*, %)	2 (3%)	5 (8%)	
Antithyroid medication use (yes/no)	13/9	20/24	0.433
Induction agent			0.473
Thiopental	30 (45%)	27 (41%)	
Propofol	3 (5%)	6 (9%)	
Surgeon experience			0.240
Resident (*n*, %)	23 (35%)	28 (42%)	
Senior (*n*, %)	10 (15%)	5 (8%)	
Estimated intraoperative blood loss (ml)^∗^	180 (200)	150 (150)	0.689
Duration of surgery (minutes)^∗^	110 (40)	110 (43)	0.508
Duration of anesthesia (minutes)^∗^	125 (48)	125 (43)	0.763

^∗^Median (interquartile range); *n* (%) = number (proportion).

**Table 2:** Recovery room vital signs between two groups.

	Treatment group (BSCPB) (*n* = 33)	Control group (*n* = 33)	*p* value
Baseline heart rate (mmhg)^∗∗^	79.06 ± 11.34	85.24 ± 10.60	0.026^#^
Baseline systolic blood pressure (mmhg)^∗^	112 (14)	118 (20)	0.073
Baseline diastolic blood pressure (mmhg)^∗∗^	70.70 ± 11.31	73.76 ± 7.88	0.207
Baseline mean arterial blood pressure (mmhg)^∗∗^	84.12 ± 11.30	85.85 ± 8.27	0.481
Baseline arterial oxygen saturation^∗^	94 (6)	94 (5)	0.806

^∗^Median (interquartile range); ^∗∗^mean ± standard deviation; ^#^statistically significant.

**Table 3:** Comparison of intraoperative total analgesia consumption between groups.

Intraoperative analgesia (mg)	Treatment group (BSCPB) (*n* = 33)	Control group (*n* = 33)	*p* value
Tramadol IV^∗^	100 (0)	100 (0)	0.504
Diclofenac IM^∗^	0 (0)	0 (75)	0.017^#^

^∗^Median (interquartile range); ^#^statistically significant; IV: intravenous; IM: intramuscular.

**Table 4:** Comparison of time to first analgesia request and total analgesia consumption between groups.

	Treatment group (BSCPB) (*n* = 33)	Control group (*n* = 33)	*p* value
Time to first analgesia request in minutes	360 (530)	180 (295)	0.006
Total analgesia consumption within 24 hours			
Tramadol, mg (IV)	0 (50)	100 (125)	0.001
Diclofenac, mg (IM)	75 (75)	75 (75)	0.775

IV: intravenous; IM: intramuscular.

## Abbreviations

ASA: American Society of Anesthesiologist
BSCPB: Bilateral superficial cervical plexus block
IASP: International Association for the Study of Pain
IM: Intramuscular
IV: Intravenous
GA: General anesthesia
MAP: Mean arterial pressure
NRS: Numeric rating scale
NS: Not statistically significant
NSAIDs: Nonsteroidal anti-inflammatory drugs
PACU: Postanesthesia care unit
PONV: Postoperative nausea and vomiting
PR: Pulse rate
PCA: Patient-controlled analgesia
SBP: Systolic blood pressure
SCM: Sternocleidomastoid muscle
SCPB: Superficial cervical plexus block
SD: Standard deviation
SPO2: Saturation
VAS: Visual Analogue Scale
WHO: World Heath Organization.
